# Function prediction from networks of local evolutionary similarity in protein structure

**DOI:** 10.1186/1471-2105-14-S3-S6

**Published:** 2013-02-28

**Authors:** Serkan Erdin, Eric Venner, Andreas Martin Lisewski, Olivier Lichtarge

**Affiliations:** 1 Department of Molecular and Human Genetics, Baylor College of Medicine, One Baylor Plaza, Houston, Texas 77030, USA; 2 Computational and Integrative Biomedical Research Center, Baylor College of Medicine, One Baylor Plaza, Houston, Texas 77030, USA

## Abstract

**Background:**

Annotating protein function with both high accuracy and sensitivity remains a major challenge in structural genomics. One proven computational strategy has been to group a few key functional amino acids into templates and search for these templates in other protein structures, so as to transfer function when a match is found. To this end, we previously developed Evolutionary Trace Annotation (ETA) and showed that diffusing known annotations over a network of template matches on a structural genomic scale improved predictions of function. In order to further increase sensitivity, we now let each protein contribute multiple templates rather than just one, and also let the template size vary.

**Results:**

Retrospective benchmarks in 605 Structural Genomics enzymes showed that multiple templates increased sensitivity by up to 14% when combined with single template predictions even as they maintained the accuracy over 91%. Diffusing function globally on networks of single and multiple template matches marginally increased the area under the ROC curve over 0.97, but in a subset of proteins that could not be annotated by ETA, the network approach recovered annotations for the most confident 20-23 of 91 cases with 100% accuracy.

**Conclusions:**

We improve the accuracy and sensitivity of predictions by using multiple templates per protein structure when constructing networks of ETA matches and diffusing annotations.

## Introduction

Predicting function for the ever-increasing number of sequences and structures produced by Genomics Centers remains a major problem [1,2]. Fewer than 0.5% of current UniProt database protein annotations come from experiments [3], showing that despite recent advances in high-throughput functional screening experiments, these are far from being sufficiently scalable to characterize protein function on a massive scale. In the foreseeable future therefore computational annotation of protein function will remain essential.

The computational tools for this purpose [4] are based on sequence or structure [5]. Here, we focus on the latter, with the rationale that structure is more conserved than sequence during evolutionary divergence [6]. As a result, any global (described by CATH [7] or SCOP [8] codes) similarities that exist between structures may indicate functional similarities that are not recognizable from sequence comparisons alone [9]. One pitfall, however, is that proteins with the same fold can often perform unrelated functions, for example TIM Barrels [10]. Thus, fold-based prediction may not be specific for a majority of functions [11]. Another pitfall is that some functions can be carried out by different folds. For example, subtilisin and trypsin are both are serine proteases with different structure. However, both share an identical catalytic triad of His-Ser-Asp [12], suggesting that functional similarity may be detected from local similarity of just a few key catalytic residues [13-16].

This observation spurred template approaches on the hypothesis that if relatively few residues determine binding or catalytic activity, then the presence of these identical residues in identical geometries may suggest an identical function even if the structure is different [13-16]. This raises a series of challenges: to detect those key functional residues, to locate them in other structures, and to make sure that these matches are not random. The Evolutionary Trace Annotation (ETA) pipeline was developed to address each of these problems [17]. ETA generates templates using Evolutionary Trace (ET) to identify putative functional sites, and their key residues, in a protein structure [18-20]. This use of ET obviates the need for any prior information on functional mechanisms in order to build templates. These ET-based templates are then matched to other structures and matched pairs of structures are accepted when the matched sites have sufficient evolutionary and geometric similarities. The requirement for evolutionary similarity specifically ensures that these matches are at sites that are functionally important in both proteins, which decreases the likelihood that the match is due to random chance [21]. Finally, ETA transfers function between the matched proteins, from an annotated structure to an unannotated structure. A public ETA webserver is available for that purpose [22].

We systematically tested this protocol for enzymes [17,23,24] and non-enzymes [24] using Enzyme Commission (EC) numbers [25] and Gene Ontology terms [26] as functional classifications. The accuracy was 92% and 94% for enzymes and non-enzymes respectively, with sensitivity near 50% in both [23,24]. To raise sensitivity, we then pooled together all ETA matches into a network of protein structures [27] and let functional information diffuse globally within it from proteins of known function to unannotated ones. The accuracy improved to 96% at 65% coverage in a test set of 1217 Structural Genomics enzymes. Similarly, others have also used network analyses to link functional information to unknown proteins in three diverse enzyme superfamilies [28].

In this work, we aim to raise sensitivity further by allowing more than one template for each protein. A single template may on occasion fail to capture a true functional site [24], and if a structure has multiple functional sites, secondary functional annotations would be missed. To further relax our requirements, we also test smaller templates with just five residues instead of exactly six residues. In a test set of 605 Structural Genomics enzymes, the combination of these innovations increased sensitivity by 14% with a modest decrease in accuracy (5%) over the default ETA. Moreover, when we applied network diffusion, performance rose further to yield an area-under-the-curve of 0.97 regardless of the ETA variant.

## Results and discussion

**Figure 1:**
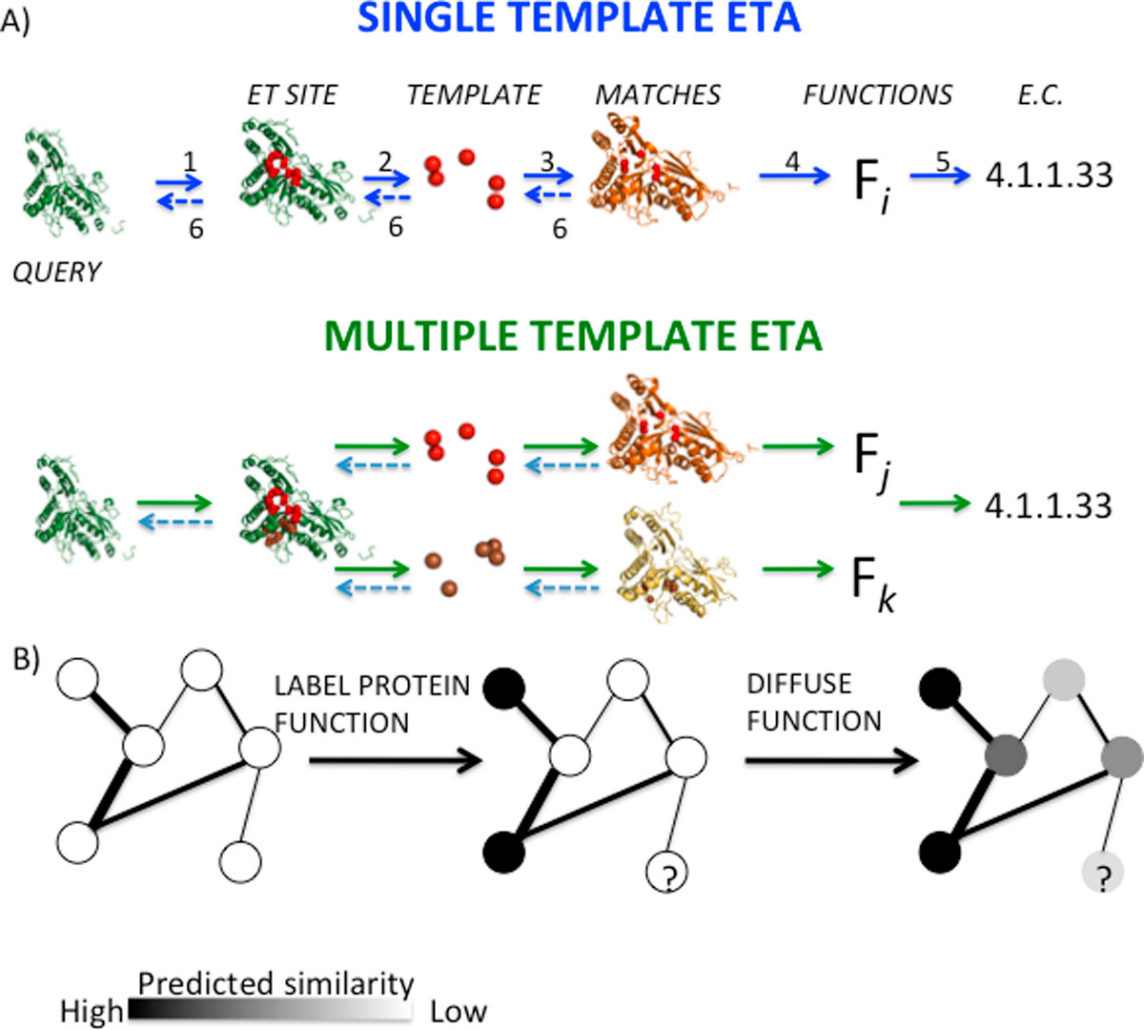
**(A) Graphical outline of Evolutionary Trace Annotation (ETA) pipeline in single template and multiple template modes**. The numbers 1,2,3,4,5,6 denote the components of ETA pipeline; Evolutionary Trace, Template Picker, Paired-distance Matching, Support Vector Machine and Reciprocity respectively. (B) Graphical outline of network diffusion method.

ETA follows an annotation strategy that consists of five steps, illustrated in both single and multiple templates ETA modes in Figure 1A, for the case of a mevalonate pyrophosphate decarboxylase from *Mus musculus *(PDB 3f0n; chain A; EC 4.1.1.33) (see Methods for details). First, ET ranks the evolutionary importance of the residues from the query protein, 3f0nA, for which the function is sought. These top-ranked ET residues usually form surface clusters, and ET identifies two significant clusters that may be functional sites: the first is {26K, 156S, 158S, 161R and 215M} and the second is {123G, 125A, 126S, 127S, 305D, 306A, 307G, 309N}. In single template mode, ETA picks template residues from the larger of the two ({305D, 307G, 123G, 126S and 127S} shown red in Figure 1A). But in multiple template mode, ETA also picks an additional five-residue template made of the entire first cluster (brown template in Figure 1A). Next, a paired-distance matching algorithm searches for geometric similarity between templates and structures from a subset of the Protein Data Bank (PDB) filtered for sequence identity and annotated with one or more known functions. These preliminary matches are filtered by a support vector machine (SVM) that selects a refined set that combines geometric similarity with ET rank similarity. To further increase specificity, ETA repeats the same steps in reverse: now templates are generated in the matched structures and searched for in the original query. Accepting only matches that fulfill such reciprocity reduces the likelihood that they arose due to random chance. In the example, ETA identifies mevalonate diphosphate decarboxylase from *Staphylococcus aureus *[29](PDB 2hk3; chain B; EC 4.1.1.33) as a reciprocal match in the single template mode, while human mevalonate diphosphate decarboxylase [30] (PDB 3d4j; chain B; EC 4.1.1.33) is added to reciprocal matches in multiple template mode. In the fifth step, ETA selects the function seen in a plurality of the target structures that matched reciprocally to the query. In our example, EC 4.1.1.33 is selected with one and two votes in single and multiple template modes, respectively.

We can extend this approach by organizing all matches into a network [27]. In this network, each protein structure is a node; each ETA match is a symmetric edge; and all known functions are treated as distinct node labels (see Figure 1B). We calculate a weighted edge from the RMSD and differences in ET scores in an ETA match [27]. Then, for each function, we label nodes based on whether they are known to have a given function, known not to have it, or whether this is unknown. We can then set up a minimization problem to find a set of diffused labels that aim to preserve the initial labeling while at the same time assigning neighboring nodes similar labels (see Methods). This process is repeated for all possible labels, *i.e*. functions, so as to yield a normalized confidence score to every possible pairing of nodes and labels, i.e. for every possible protein function prediction. We then evaluate the accuracy of the highest confidence prediction.

**Figure 2:**
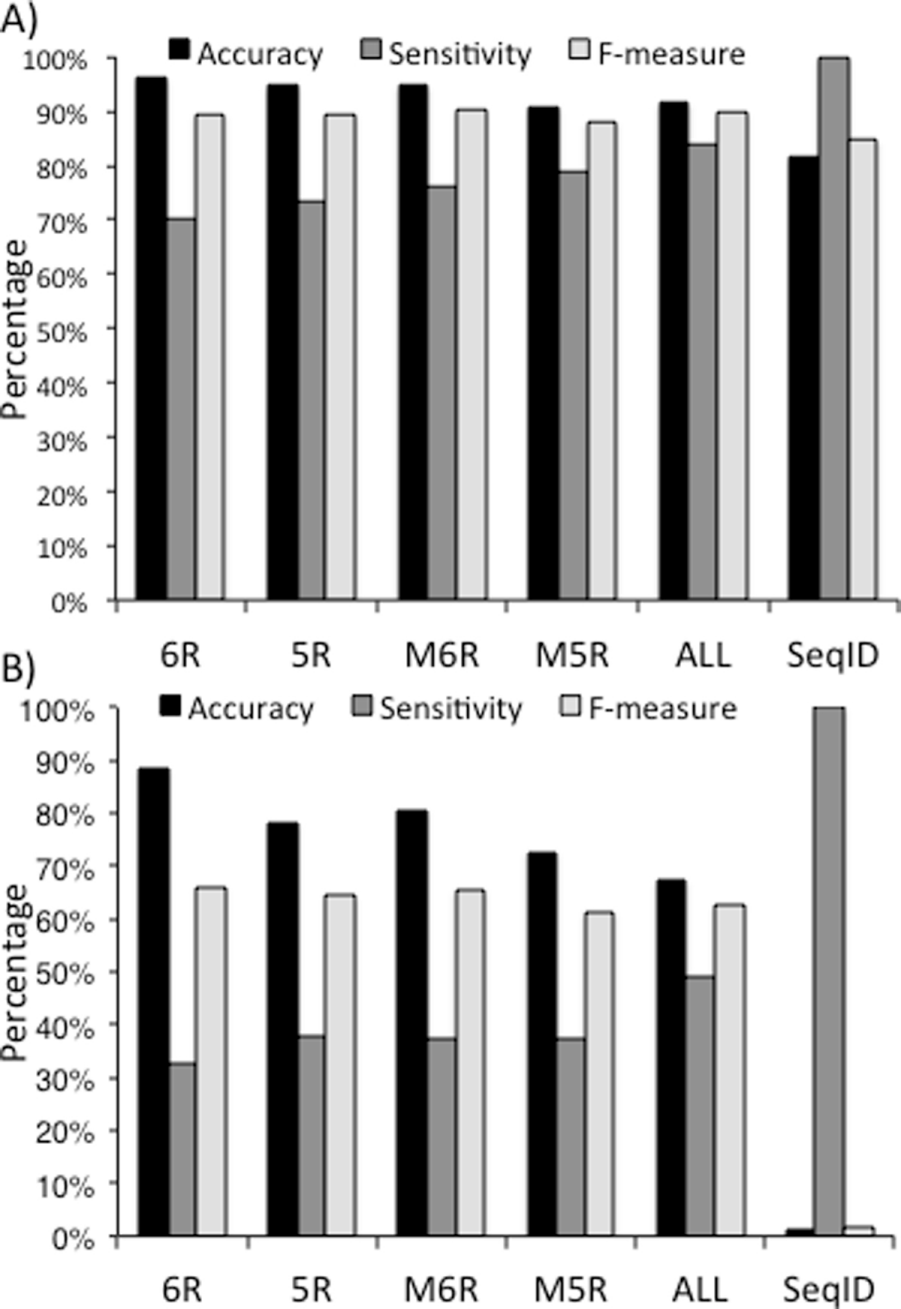
**Accuracy, sensitivity and F-measure of ETA with four template selection methods, six-residue (6R), five-residue (5R), multiple six-residue (M6R), multiple five-residue (M5R), combination of four ETA template selecting modes (ALL) and a sequence-based annotation based on sequence identity (SeqID) for (A) a test set of 605 Structural Genomics enzymes; and for (B) a non-trivial test set of 73 Structural Genomics enzymes**.

We wish to benchmark ETA's performance across four possible modes: the first selects matches based on one five-residue template per protein (5RT); the second relies instead on one six-residue template per protein (6RT); the third uses multiple five-residue templates per protein (M5RT); and the last uses multiple six-residue templates per protein (M6RT). In each mode, ETA was applied on a set of 605 enzymes with full-EC annotations (see Methods). Performance of each template selection mode and sequencebased strategy was measured in terms of accuracy, sensitivity and by the weighted mean of both with the F-measure (see Methods). The results, depicted in Figure 2A, showed that while single-templates were more accurate, multiple templates were more sensitive. Overall, it was the multiple six-residue templates that yielded the highest F-measure performance, suggesting this is the method of choice.

Next, we sought to assess performance in a subgroup of especially difficult cases. To this end, we identified 73 query proteins that share no more than 30% sequence identity with any annotated protein in the target set with the same function. The results (Figure 2B) show that smaller, multiple templates increased sensitivity with only a slight, tolerable decrease in accuracy. The best overall performance in terms of F-measure, was from six-residue templates followed by multiple six-residue template (See Figure 2B).

In order to compare each template selection mode with a sequence-based annotation strategy, we identified the closest hits from target set to each protein in both query sets based on sequence identity between the query and the target. We label this strategy SeqID. Each template selection mode outperformed SeqID in terms of accuracy and F-measure in both test sets (see Figure 2). In case of non-trivial proteins, four ETA template selection modes yielded average 0.643 in terms of F-measure versus 0.017 by the SeqID. This result shows that our template-based strategy is especially useful if the protein of interest does not have close homologs.

To test whether different template modes yield similar annotations, we evaluated the similarity of all pairs of predictions. The performance of a given template selection category was represented as a vector of functions and compared their normalized scalar product (see Methods). Tables 1A and 1B reveal from these products that each method has a number of uniquely accurate predictions, suggesting that their combination might achieve perform even better. As expected, the methods with higher accuracy have lower sensitivity (see Figure 2A).

**Table 1:** Prediction similarity of four ETA methods with one another (A) in cases of 605 Structural Genomics enzymes, and (B) in cases of 73 non-trivial Structural Genomics enzymes.

A)		**6R**	**5R**	**M6R**	**M5R**
	
	**6R**	1.00	0.93	0.92	0.83
	**5R**		1.00	0.88	0.88
	**M6R**			1.00	0.88
	**M5R**				1.00
B)		**6R**	**5R**	**M6R**	**M5R**
	
	**6R**	1.00	0.82	0.93	0.70
	**5R**		1.00	0.81	0.82
	**M6R**			1.00	0.74
	**M5R**				1.00

This led us to an iterative annotation strategy that applies first the template selection method with the best accuracy, followed in further rounds by the next best, and so on. The methods were ordered by their accuracies as 6R > 5R > M6R > M5R following Figure 2A. As a result, sensitivity rose to 83.8% and accuracy to 91.4%. In Figure 2B, the accuracy order is 6R > M6R > 5R > M5R. This strategy achieved accuracy of 67.4% and sensitivity of 49.2% in this particularly challenging test set.

**Figure 3:**
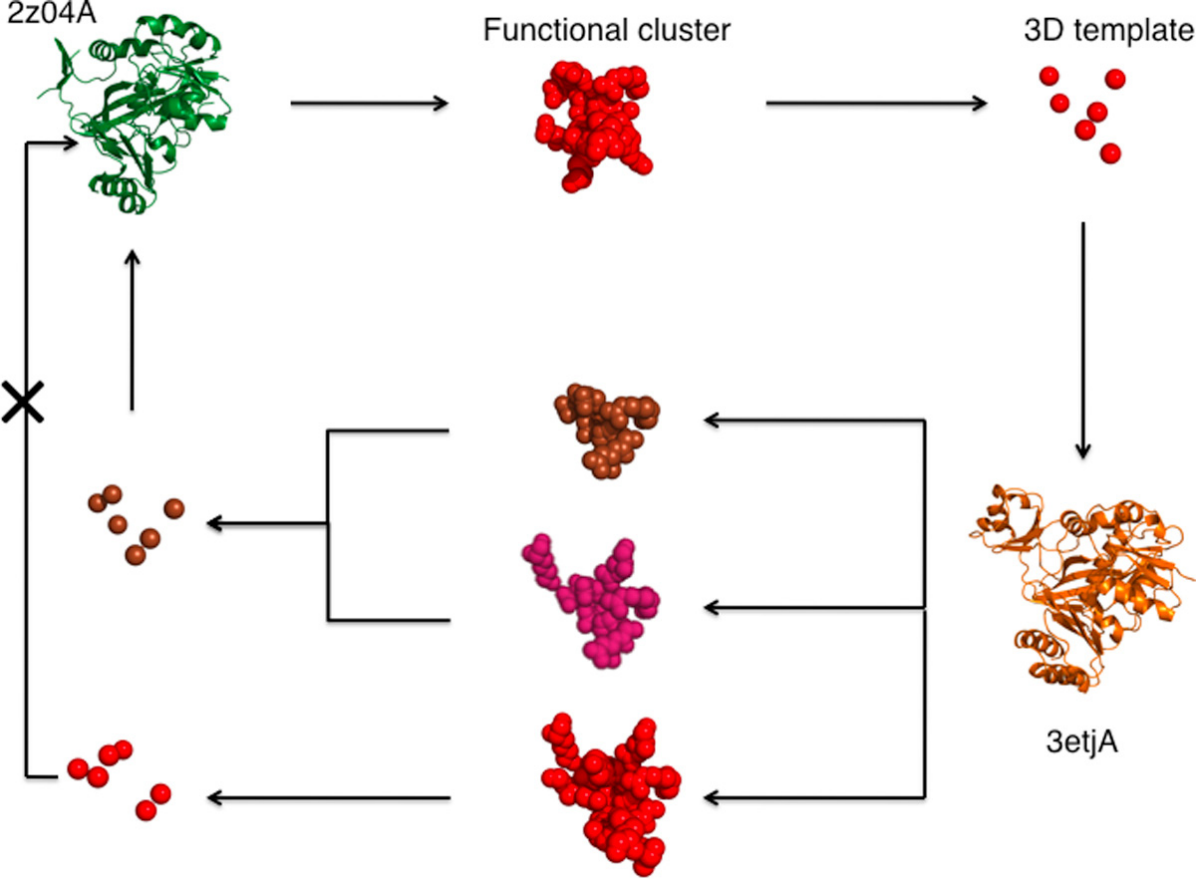
**Graph of ETA matches between 2z04A and 3drjA based on six-residue and multiple six-residue templates**. Red clusters and red templates are identified in single template mode, while brown and purple clusters, and brown templates are identified in multiple template mode.

An example, in Figure 3, illustrates how ETA in multiple templates per protein mode recovered annotations missed by ETA with a single template per protein. ETA generated a six-residue template, {257N, 256H, 260H, 254R, 237E, 250E}, from the Phosphoribosylaminoimidazole carboxylase ATPase subunit from *Aquifex Aeolicus *(PDB 2z04; chain A) and matched it to a N5-carboxyaminoimidazole ribonucleotide synthetase from *Esherichia Coli *with 30% sequence identity (PDB 3etj; chain A) [31]. However, the reciprocal six-residue template in 3etjA, which is {126Y, 127D, 128G, 245N, 244H} generated from ET cluster {51E, 120K, 126Y, 127D, 128G, 226E, 237N, 238E, 242R, 244H, 245N, 305Y, 307K, 314K}, could not be matched significantly back to the query 2z04A, and as a result there was no prediction in a single template mode. In multiple template mode, however, ET identified two other subclusters in 3etjA {226, 237, 238, 242, 244, 245} (shown in brown) and 51, 120, 226, 237, 238, 242, 244, 245} (shown in purple) and ETA accordingly generated an additional reciprocal six-residue template {226E, 237N, 238E, 242R, 244H, 245N}, which did match to 2z04A and thereby led to the correct prediction.

**Figure 4:**
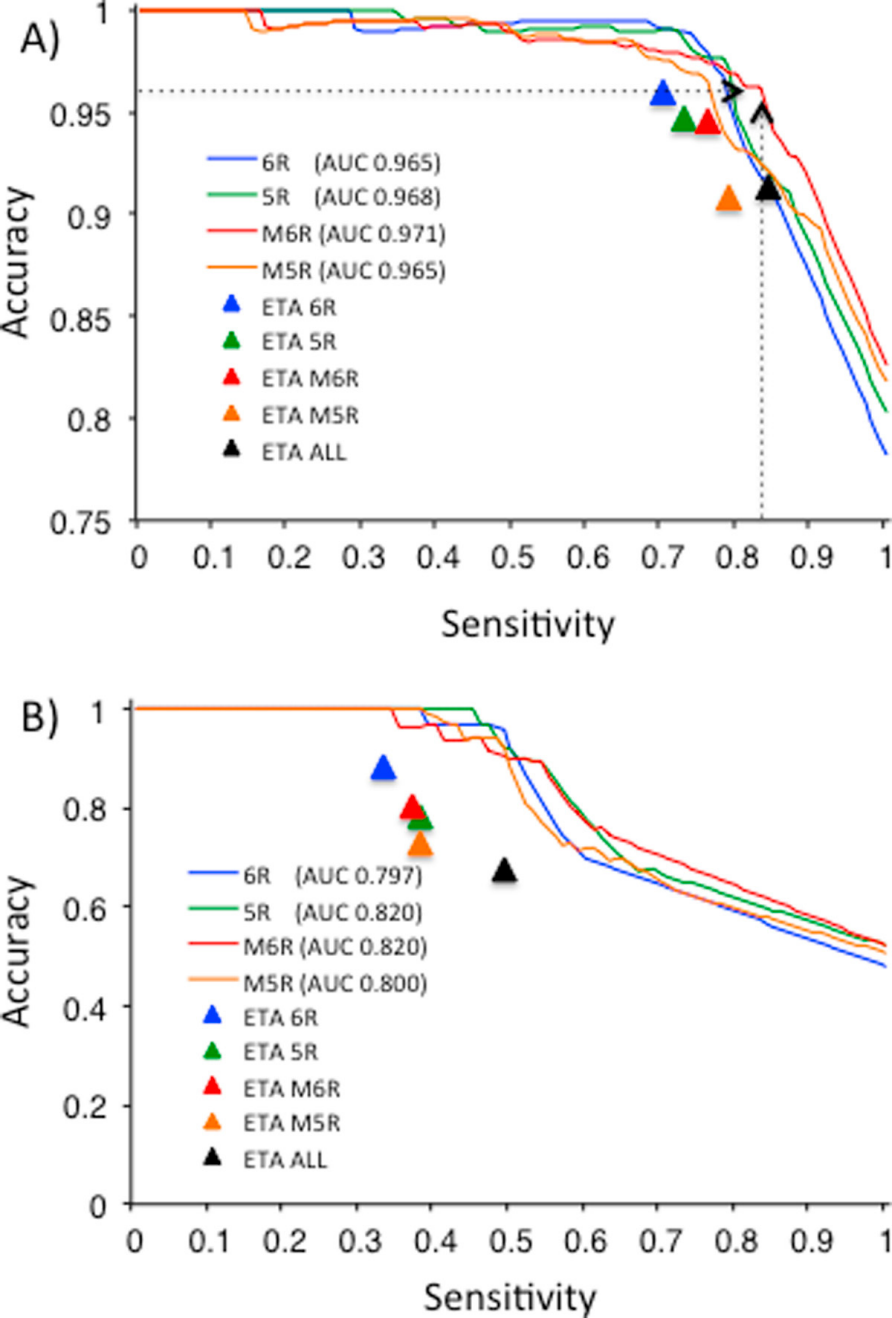
**Accuracy versus sensitivity graph of network diffusion method for four different ETA network for (A) 605 Structural Genomics enzymes; and for (B) 73 non-trivial Structural Genomics enzymes**. The numbers inside the parentheses show the area under curve (AUC) for each curve. Dashed line indicates the performance of network diffusion method based on multiple six-residue templates.

In order to further assess performance, we constructed networks of ETA matches from each of the modes of ETA described above. Competitive diffusion was then carried out as described previously [27] in order to draw annotation from the global distribution of all matches among all query proteins and all proteins with known functions. The results suggest that the predictive power of the network makes up for the disadvantages of each individual method since all of the template methods perform nearly equally well. In the 605 protein benchmark test set, the area under the accuracy-sensitivity receiver operator curves were essentially identical at 0.971, 0.968, 0.965, and 0.965 for multiple six-residue templates, five-residue templates, six-residue templates, and multiple five-residue templates, respectively (see Figure 4A). In more detail, however, some slight differences emerge. At 95% accuracy, the network built from multiple six-residue templates per protein has 4% better sensitivity of (84 vs 80%) over six-residue single template networks, accounting for 24 additional true positives. This improvement was also observed in the benchmark of 73 proteins with less than 30% sequence identity to any true annotated matching protein, as shown in Figure 4B.

To evaluate the ability of our confidence score to identify accurate predictions, we plotted cumulative accuracy against confidence (z-score) (Additional file 1). The predictions separate into three regions: over a confidence value of 2.0, predictions are nearly 100% accurate across all ETA modes. In the range between 2.0 and 0.5, accuracy begins to drop: In this range, predictions are 96% accurate regardless of ETA variant. Finally, below 0.5, accuracy declines steeply, with 39%, 22%, 20% and 12% accuracy for multiple five-residue, multiple six-residue, five-residue and six-residue networks, respectively.

**Figure 5:**
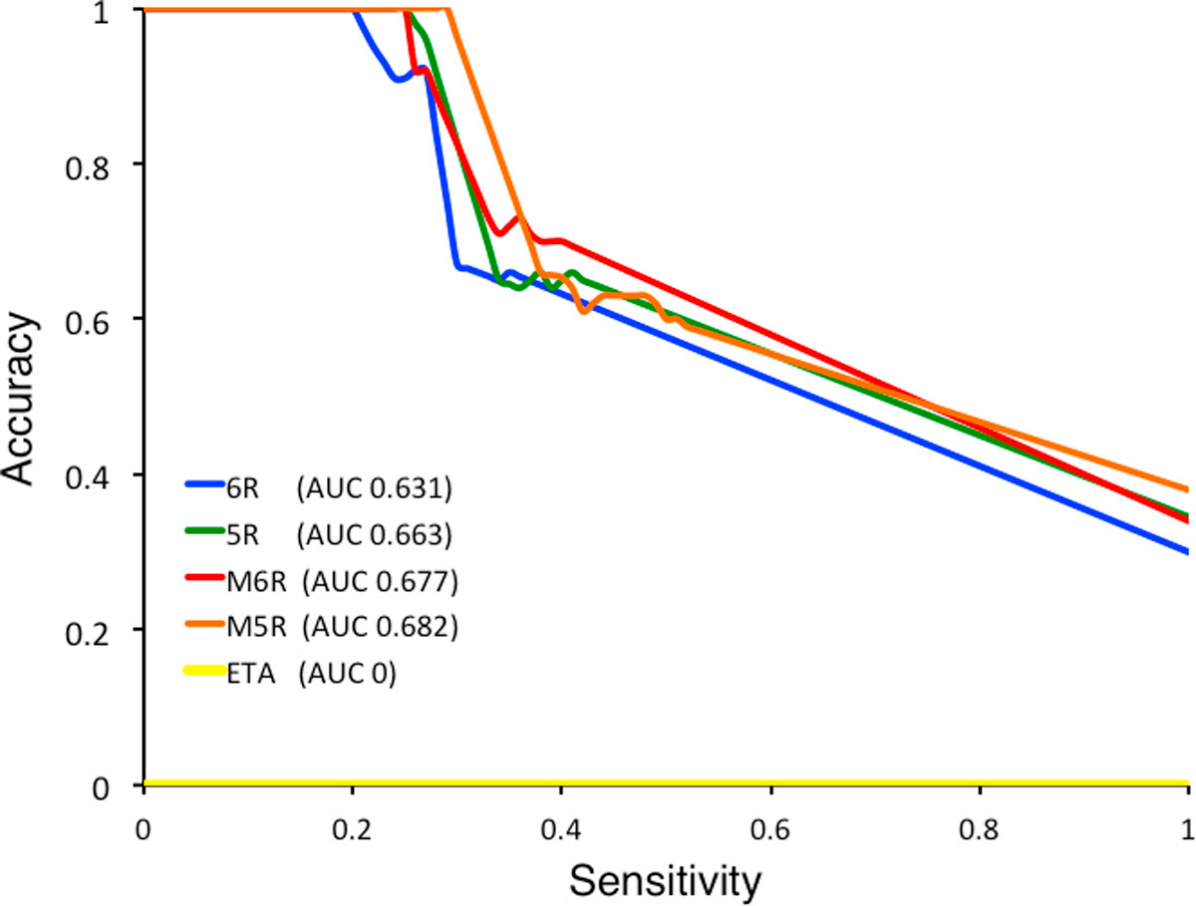
**Accuracy versus sensitivity graph of network diffusion method for four different ETA networks based on six-residue (6R), five-residue (5R), multiple six-residue (M6R) and multiple five-residue templates (M5R) for 91 Structural Genomics enzymes with no ETA prediction with any template methods**. The numbers inside the parentheses show the area-under-curve for each curve.

Direct comparisons with default ETA (see the triangles in Figure 4) illustrate that incorporating global information extends the accuracy and sensitivity of predictions over ETA alone. This can also be seen by focusing on the network predictions for the 91 protein structures for which ETA alone had none. As depicted in Figure 5, all of the ETA networks were able to make predictions for these 91 proteins with those based on multiple templates yielding the best performance with around 0.68 area-under-curve (AUC). However, all of the ETA networks' accuracy rose up to 100% at around 21-29% sensitivity, which accounted for 20-23 cases depending on the network (see Figure 5).

**Figure 6:**
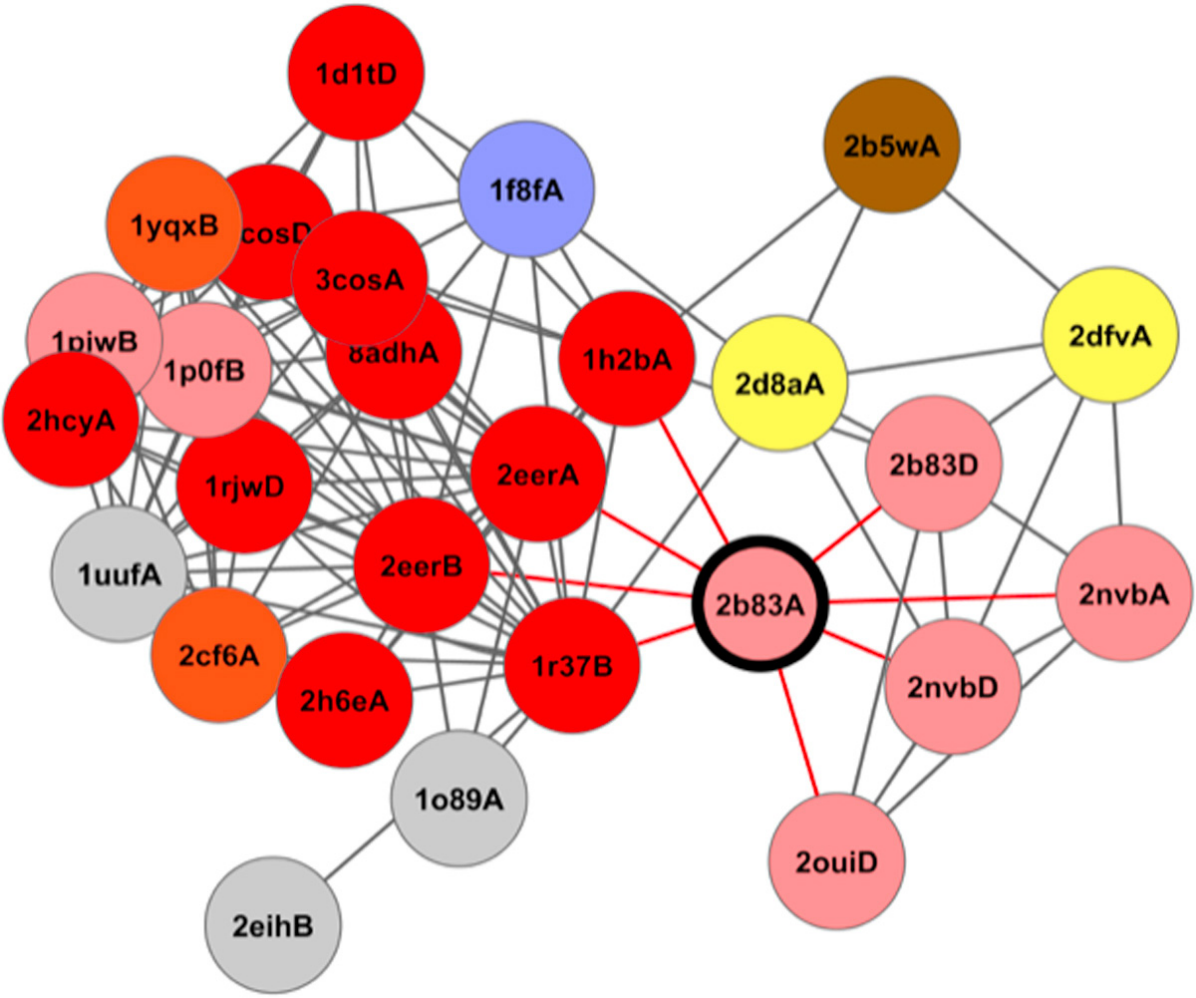
**View of a sub-network around 2b83A (center, dark border)**. Red links are direct matches based multiple six-residue ETA and grey links are secondary connections. Proteins are represented by circles of different colors that denote the enzymatic function (red: EC 1.1.1.1, pink: EC 1.1.1.2, orange: EC 1.1.1.95, brown: EC 1.1.1.47, yellow: EC 1.1.1.103, lavender: EC 1.1.1.90). The network diffusion model is able to make a correct prediction in this case due to the weight of the edges and proximity of correct functional labels in the network to 2b83A.

The gene *ahd *from the bacterium *clostridium beijerinckii *(PDB 2b83; chain A) [32]highlights the benefit of ETA networks. ETA makes no prediction because of a four apiece tie between matches to NAD-dependent alcohol dehydrogenase activity (EC 1.1.1.1) and to NADP-dependent alcohol dehydrogenase activity (EC 1.1.1.2.). But, when edge weights are taken into account by the network, the stronger connectivity to nodes labelled with EC 1.1.1.2 break the tie and give the edge, correctly, to this annotation. The confidence score is moderate (0.5), reflecting the difficulty of disentangling this dense cluster of matches (Figure 6).

## Conclusion

This work aimed to increase the sensitivity of function annotation in protein structures through alternative ET-based template matching strategies. We found that using more than one template per protein raised sensitivity by 6-9% at a cost of 1-5% in accuracy. Overall, the use of multiple templates did yield a better annotation performance as shown by the increase in F-measure. Furthermore, network diffusion based on multiple-template matches outperformed single template-based network diffusion with 3-8% increase in AUC in recovery of the functional classification of cases where ETA completely failed. Interestingly, the simultaneous use of multiple templates arising from all of the structures under consideration was always best, and robust enough to be insensitive to the size or number of templates per protein. Thus, when functional labels are diffused over a network of ETA matches, the result plateaus at a very high AUC value between 0.96 and 0.97. Most usefully, these experiments define prediction confidence thresholds that distinguished reliable prediction, from those less so, and those that likely to be no better than chance. The results show that among proteins with no ETA prediction, diffusion over a network can add novel predictions. More broadly, this strategy of competitive annotation diffusion over ETA-networks should help accurate large-scale annotation of the structural proteome.

## Methods

Data Sets: Our query test set contains 605 enzymes from Structural Genomics centers that cover 348 distinct full-EC numbers. Each pair of proteins in this set share less than 90% sequence identity with one another. Non-trivial test set constitute 73 proteins of the query set. These proteins share at most 30% sequence identity with any protein of the same function at the full-EC level in the target set. Target proteins are selected from 17824 2008PDB90 [33] proteins with the criteria that their truncation ratio greater than 0.95. Truncation ratio is defined to be the ratio of the number of amino acids in the structure to the actual sequence length of the protein. Truncation ratio shows how much of the protein is solved in the structure. 8537 proteins hold this criterion and 3082 of them carry full-EC annotations that cover 1190 distinct catalytic function in the databases of Uniprot/SwissProt/TrembL [3] and PDB [33]. Additional file 2 shows the composition of these three sets in terms of PFAM protein families [34]. In doing so, only three and two proteins had unassigned PFAM accessions from 605-query test set and 73-query test set respectively, while 3082-protein target set had only 14 proteins with no PFAM accessions. The most represented protein families in 605-query test set and 3082-protein target set were Aminotran_1_2 (PFAM:PF00155) with 13 proteins and Adh_short (PFAM:PF00106) with 60 proteins respectively. Non-trivial query set with 73 proteins had three most represented protein families Hydrolase_3 (PFAM:PF08282), Pyr_redox_2 (PFAM:PF07992) and DeoC(PFAM:PF01791), each were associated with three proteins. Therefore, the portions of overrepresented protein families in 605-protein and 73-protein query test sets and 3082-protein target set are 2%, 4% and 2% respectively.

Evolutionary Trace: In this work, we used the real-valued version of Evolutionary Trace (ET) algorithm whose detailed description can be found elsewhere [19,35]. Briefly, ET algorithm first identifies homologous sequences for a query protein by performing BLAST [36] searches over NCBI Entrez non-redundant protein sequence database. The identified BLAST hits shared minimum 20% and maximum 95% sequence identity with the query sequence. Next, ClustalW [37] generates multiple sequence alignment for the identified sequence hits for the query protein. Further, a phylogenetic tree is obtained by using UPGMA algorithm [38]. Finally, ET algorithm calculates the evolutionary ranks by employing a combined method of integer ET [18] and Shannon Entropy [39] that quantifies correlation of variations in multiple sequence alignment with branching in phylogenetic tree for each amino acid in the query sequence.

Template selection: In this work, we used five and six-residue templates relying on a previous study showing that these sizes maximized accuracy and sensitivity [17]. Template creation was described in details elsewhere [17]. Briefly, template picker algorithm performs the following steps: ETA first applies real-valued version of ET algorithm (see the above section) to assign evolutionarily importance ranks to the amino acid residues in a protein structure of interest and sorts clusters of important residues according to their evolutionarily importance rank from the lowest (the most important) to the highest (the least important). The cluster size increases as the rank increases. In case of single templates of five or six residues, ETA first identifies the cluster of at least 11 important surface residues whose surface accessible area is greater than 2Å^2^, which is computed by DSSP [40]. Each residue is represented by its C_α_'s coordinates. Template selection algorithm further identifies the center of mass (CM) of the chosen clusters and the closest best ranked-amino acid as the first residue of the template. In order to identify the other residues of the template, the algorithm iteratively selects other most important residues that are closer to the midpoint between the CM of cluster of selected residues in the previous iteration and the CM of the chosen cluster. Residue positions in the templates are labeled by both original residue types in the structure and any non-gapped combination that is at least seen twice in the multiple sequence alignment. In order to generate multiple templates, ETA first identifies a cluster containing at least 11 important surface residues as the first cluster. Next, ETA identifies other clusters, if any, that do not completely overlap with the first cluster, and that contain surface residues with better evolutionary importance ranks than those in the first cluster. Additional templates from those clusters were generated by following the same steps as in case of single templates. Therefore, the number of templates for a given protein was dictated by the number of identified distinct clusters of evolutionary important surface residues for that protein. For example, if there is no other distinct cluster than the cluster chosen for single templates, the template selection algorithm yields only a single template.

Template searching: ETA utilizes paired-distance matching (PDM) algorithm to probe geometric similarities between query templates and other structures. In doing so, PDM first identifies all the residues that are identical to the first residue types of the query template in the other structure. In the next iteration, PDM identifies the residues that are identical to the type of second template residue. PDM retains only the pair of residues whose paired-distance is within 2.5Å with the paired distance of first and second template residues. Further, residues that are identical to the type of third template residue are identified. PDM again applies distance constraint of 2.5 Å upon comparison of combination of all possible-paired distances in order to select the third residue. In further iterations, these steps are repeated to select other residues in the target structure. Each match is assigned with a value of root mean square deviation (RMSD) to quantify the geometric similarity.

Match filtering: In the next round, ETA eliminates self-matches and matches with RMSD greater than 2 Å. The remaining matches are fed into the Support Vector Machine (SVM) that is trained for enzymes [23]. Each match is represented by either six or seven dimensional vectors depending on the template size. One dimension is for geometric similarity whereas others quantify evolutionary similarity. The latter is computed as the absolute value of percentile-rank differences of a query template residue and matched residue in the target structure. We use the same six-residue SVM for five-residue ETA by constructing a virtual sixth residue as the average of the other five positions. In the end, SVM filters the significant matches.

Reciprocal match: Geometric and evolutionary similarity is probed by both template(s) from query protein onto target structures and templates from target structures onto the query protein. Reciprocal matches constitute the intersection of significant matches selected by SVM in both directions.

Function prediction: In the final round, ETA assigns votes to the functions of unique reciprocal matches and thereby suggests the function with the highest vote as a predicted function. If a particular protein is identified as a significant match several times for a query protein, its function gets only one vote.

Performance measures: We used accuracy, sensitivity and F-measure to assess ETA's performance, which are defined as follows: Accuracy = TP/(TP+FP) and Sensitivity=(TP)/(TP+FN), where TP is true positive, both known and predicted function agree at the fourth EC level, FP is false positive, predicted and known function does not agree at the fourth EC level, and FN is false negative, which represents the cases with no prediction. F-measure = (1+β^2^). accuracy. sensitivity/(β^2 ^. accuracy + sensitivity), where β equals 0.5 to put more emphasis on accuracy.

Sequence identity: In order to calculate the sequence identity between two proteins, we first aligned their sequences by ClustalW [37]. Sequence identity is defined to be the ratio of the number of aligned identical residues in both sequences to the total number of aligned residues.

Prediction similarity: ETA's performance at a given template selection category was expressed in a vector form of length N, where N is the size of query test set. Each element of the vector represents a particular query protein, which is labeled by either correct, incorrect or no prediction tags. Similarity between performances of category 1 and 2 is assessed by $S\left(1,2\right)=\overset{N}{\underset{i=1}{\sum }}\frac{P_{i}^{1}•P_{i}^{2}}{N}$, where $P_{i}^{1}$ denotes the type of tag attached to the i^th ^protein in the query set based on category 1 annotation scheme. Then, $P_{i}^{1}•P_{i}^{2}=1$ if $P_{i}^{1}=P_{i}^{2}$, else $P_{i}^{1}•P_{i}^{2}=0$.

ETA networks: ETA networks are constructed in three parts: query-query, query-target and target-target. In each part, an ETA variant is used to identify significant reciprocal matches. For example, in the query-query part, ETA identifies a reciprocal match for each protein in the query set from another non-self match protein of query set. Query-target part is default ETA protocol. In the target-target part, ETA assigned reciprocal matches for each protein in the target set from another non-self match protein in the target set.

Once detected, weighted edges in the networks were calculated from an ETA match with:

$$1/2\left[\left(rmsd-\mu_{rmsd}\right)/\sigma_{rmsd}+\left(ETScore-\mu_{ETScore}\right)/\sigma_{ETScore}\right]$$

ETA provides the rmsd and ETScore which collectively describe the quality of a template match. An ETScore reflects the total difference between the evolutionary score of matched residues. The rmsd is the geometric difference between the template and the matching structure. μ_rmsd _is the average rmsd for all matches in the network, σ_rmsd _is the standard deviation for all rmsds in the network, μ_ETScore _is the average ETScore for all matches in the network and σ_ETScore _is the standard deviation of all ETScores in the network.

We store the resulting network in an adjacency matrix, W. If there are n nodes, W is n by n and W_ij_ is set to the weight calculated above if a match is detected between protein i and j, or set to 0 if there is no such match. We then create an n-dimensional label vector y for a particular function present in our network by setting y_i_, 0 ≤ i < n to 1 if node i is known to have that function, to -1 if it is known to not have that function, or to 0 if the protein has unknown function (or is a member of the test set). We use these elements to pose an optimization problem:

$H=\underset{i}{\sum }{\left(f_{i}-y_{i}\right)}^{2}+\alpha \underset{i,j}{\sum }w_{ij}{\left(f_{i}-f_{j}\right)}^{2}$ The n dimensional vector f will hold predicted functional scores after diffusion. The first term penalizes nodes which deviate highly from their initial label - encouraging the system to keep the initial knowledge. The second term penalizes neighbors that have different labels according to the weight of their connection, encouraging functions to propagate through the network. The parameter α trades-off these two conditions. This can be efficiently solved as follows: f = (I + αL)y where L = D - W is the Laplacian matrix (D is the diagonal matrix, D_ii _= Σ_j _w_ij_) [41]. We repeat this process for every function represented in the network and compare the resulting values in f for each node with unknown function. To do this we normalize the values in f across all nodes with unknown function (those entries in y initially set to 0) by taking z = (f_i _- f_μ_)/f_σ _where f_μ _is the average f_i _across all proteins with unknown function and f_σ _is the standard deviation of f_i _across all proteins with unknown function. This results in a z score, which we use as a measure of confidence. The function with the largest z score at for node i becomes our prediction. The Cytocape plugin for ETA networks is available at http://mammoth.bcm.tmc.edu/networks/[42]. Multiple template and five-residue template extension will be incorporated into http://mammoth.bcm.tmc.edu/eta/.

## Competing interests

The authors declare that they have no competing interests.

## Authors' contributions

SE, EV and AML developed the methods. SE and EV collected and analyzed the data. SE and OL led the research design. The manuscript was jointly written by SE and OL with contributions from all authors.

## Supplementary Material

Additional file 1 **Graph of accuracy versus confidence for global diffusion over a network of ETA's six-residue templates**. The vertical axis shows the cumulative accuracy of cases at a given confidence score and above Click here for file

Additional file 2 **(A) Bar charts showing the number of PFAM accession codes that are associated with the number of proteins in 605-protein query test set and 73-protein query test set (non-trivial test set) in black and gray respectively**. (B) Bar chart showing the number of PFAM accession codes that are associated with the number of proteins in 3082-protein target set. Click here for file

## Declarations

This article has been published as part of *BMC Bioinformatics *Volume 14 Supplement 3, 2013: Proceedings of Automated Function Prediction SIG 2011 featuring the CAFA Challenge: Critical Assessment of Function Annotations. The full contents of the supplement are available online at URL. http://www.biomedcentral.com/bmcbioinformatics/supplements/14/S3.
