# Supplementary figures and images for: Function prediction from networks of local evolutionary similarity in protein structure
Source: BMC Bioinformatics. 2013 Feb 28;14(Suppl 3):S6. doi: 10.1186/1471-2105-14-S3-S6 (PMC3584919; doi:10.1186/1471-2105-14-S3-S6)

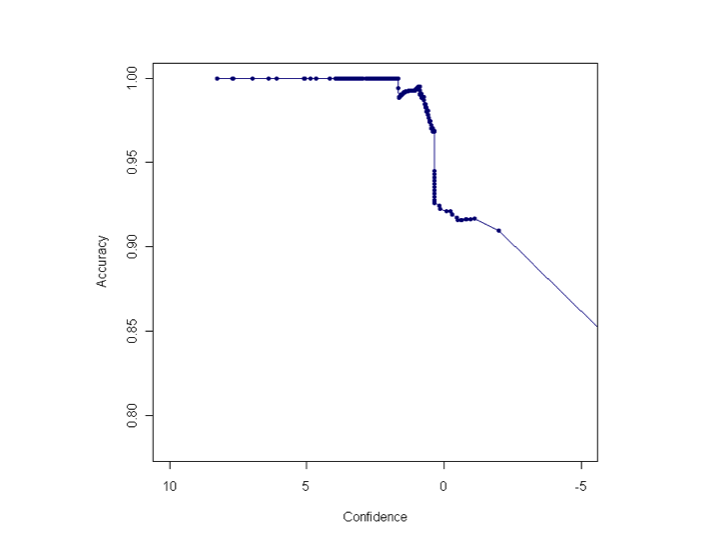

Supplement: Additional file 1 — Graph of accuracy versus confidence for global diffusion over a network of ETA's six-residue templates. The vertical axis shows the cumulative accuracy of cases at a given confidence score and above [file 1471-2105-14-S3-S6-S1.png]

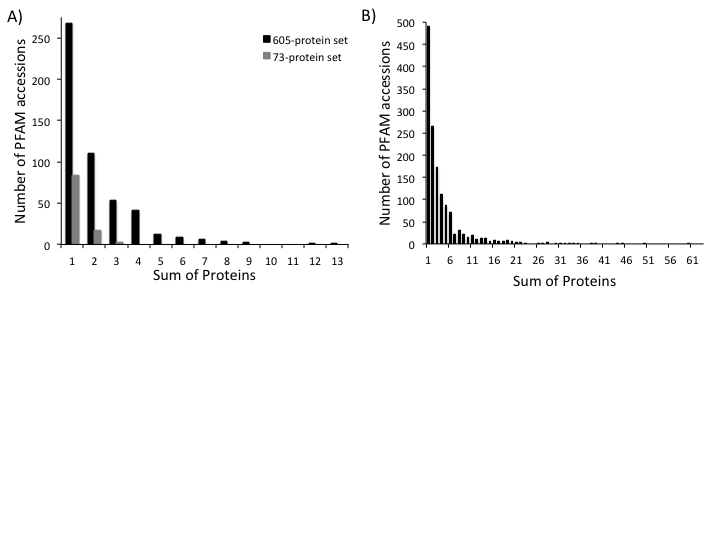

Supplement: Additional file 2 — (A) Bar charts showing the number of PFAM accession codes that are associated with the number of proteins in 605-protein query test set and 73-protein query test set (non-trivial test set) in black and gray respectively. (B) Bar chart showing the number of PFAM accession codes that are associated with the number of proteins in 3082-protein target set. [file 1471-2105-14-S3-S6-S2.png]
